# PrP^C^ Aptamer Conjugated–Gold Nanoparticles for Targeted Delivery of Doxorubicin to Colorectal Cancer Cells

**DOI:** 10.3390/ijms22041976

**Published:** 2021-02-17

**Authors:** Gyeongyun Go, Chang-Seuk Lee, Yeo Min Yoon, Ji Ho Lim, Tae Hyun Kim, Sang Hun Lee

**Affiliations:** 1 Department of Biochemistry, Soonchunhyang University College of Medicine, Cheonan 31151, Korea; ggy0227@naver.com; 2 Department of Biochemistry, BK21FOUR Project2, College of Medicine, Soonchunhyang University, Cheonan 31151, Korea; wlenfl1@naver.com; 3 Department of ICT Environmental Health System, Graduate School, Soonchunhyang University, Asan 31538, Korea; eriklee0329@sch.ac.kr; 4 Medical Science Research Institute, Soonchunhyang University Seoul Hospital, Seoul 04401, Korea; yoonboo15@naver.com; 5 Department of Chemistry, Soonchunhyang University, Asan 31538, Korea

**Keywords:** PrP^C^, PrP^C^ aptamer, colorectal cancer, gold nanoparticle, doxorubicin, drug delivery

## Abstract

Anticancer drugs, such as fluorouracil (5-FU), oxaliplatin, and doxorubicin (Dox) are commonly used to treat colorectal cancer (CRC); however, owing to their low response rate and adverse effects, the development of efficient drug delivery systems (DDSs) is required. The cellular prion protein PrP^C^, which is a cell surface glycoprotein, has been demonstrated to be overexpressed in CRC, however, there has been no research on the development of PrP^C^-targeting DDSs for targeted drug delivery to CRC. In this study, PrP^C^ aptamer (Apt)-conjugated gold nanoparticles (AuNPs) were synthesized for targeted delivery of Dox to CRC. Thiol-terminated PrP^C^-Apt was conjugated to AuNPs, followed by hybridization of its complementary DNA for drug loading. Finally, Dox was loaded onto the AuNPs to synthesize PrP^C^-Apt-functionalized doxorubicin-oligomer-AuNPs (PrP^C^-Apt DOA). The PrP^C^-Apt DOA were spherical nanoparticles with an average diameter of 20 nm. Treatment of CRC cells with PrP^C^-Apt DOA induced reactive oxygen species generation by decreasing catalase and superoxide dismutase activities. In addition, treatment with PrP^C^-Apt DOA inhibited mitochondrial functions by decreasing the expression of peroxisome proliferator-activated receptor gamma coactivator 1-alpha, complex 4 activity, and oxygen consumption rates. Compared to free Dox, PrP^C^-Apt DOA decreased proliferation and increased apoptosis of CRC cells to a greater degree. In this study, we demonstrated that PrP^C^-Apt DOA targeting could effectively deliver Dox to CRC cells. PrP^C^-Apt DOA can be used as a treatment for CRC, and have the potential to replace existing anticancer drugs, such as 5-FU, oxaliplatin, and Dox.

## 1. Introduction

Colorectal cancer (CRC) is classified as one of the four major cancers, along with lung, breast, and prostate cancers [1]. In 2020, the total number of people diagnosed with CRC was estimated to be 147,950, which accounts for 8.2% of all cancers in the United States [1]. Chemotherapy is primarily used to kill the CRC cells remaining after surgery or for symptom relief in the later stages of cancer. Fluorouracil (5-FU) is mainly used as an anticancer agent for CRC, but it shows a low response rate of approximately 20% [2]. Oxaliplatin is also used for CRC treatment. Oxaliplatin is known to be effective in colorectal cancer cells which are resistant to other platinum-based anticancer drugs. In particular, oxaliplatin shows synergistic effects through combination treatment with 5-FU [3], but the response rate for each patient varies, from 20 to 50% [4,5,6]. Doxorubicin (Dox) is an anticancer agent that is most commonly used in chemotherapy, and known to bind to deoxyribonucleic acid (DNA), to inhibit DNA synthesis and induce apoptosis. Compared to other anticancer drugs, Dox has the advantage of being relatively cheap; however, CRC patients display resistance to it, Dox induces adverse effects including vomiting, hair loss, and cardiotoxicity [7]. To overcome these challenges, there is a need for safe and efficient drug delivery systems (DDSs) for Dox.

Various DDSs, such as micelles [8,9], superparamagnetic iron oxide nanoparticles [10,11], quantum dots [12,13], dendrimers [14], exosome mimetics [15,16], viral nanoparticles [17], and gold nanoparticles (AuNPs) [18,19] are being actively developed. Among these, AuNPs have several advantages as anticancer DDSs. First, AuNPs can be synthesized in various sizes and shapes. In addition, AuNPs can easily be modified through surface chemistry to display various types of molecules on the surface, which results in the functionalization of the AuNPs. As AuNPs are known to be potentially toxic, several studies have been conducted to overcome this issue through surface modification. Various biological materials, including DNA [20], polyethylene glycol (PEG) [21,22], polyamidoamine [23], cell-penetrating peptides, and chitosan [24,25] have been used to enhance the biocompatibility of AuNPs. Furthermore, nucleic acid components can also be used to enhance drug loading. For example, oligonucleotide-coated AuNPs can be loaded with the DNA-intercalating drug Dox with high efficiency [18,19].

The cellular prion protein (PrP^C^) is a glycophosphatidylinositol-anchored cell surface protein that is associated with diverse cellular functions, including stress protection and cellular differentiation [26]. Recently, several studies have shown that PrP^C^ is highly expressed in various types of cancers, including gastric [27], pancreatic [28], breast cancers, and CRC [29,30]. Furthermore, it has been reported that PrP^C^ promotes cancer progression by enhancing cancer cell proliferation, metastasis, and drug resistance. These results suggest that PrP^C^ is a promising therapeutic target for cancer [29,30]. In this study, we hypothesized that an efficient targeted DDS could be developed for CRC treatment by targeting PrP^C^. In a previous study, we developed AuNPs coated with Dox-loaded oligonucleotides (Dox-oligomer-AuNP, DOA). In this study, we synthesized an oligonucleotide with an aptamer (Apt) sequence to target PrP^C^, which was then used to fabricate the PrP^C^-targeted Dox DDS (PrP^C^-Apt-functionalized doxorubicin-oligomer-AuNPs (PrP^C^-Apt DOA)) for CRC treatment. We evaluated the efficacy of PrP^C^-Apt DOA by determining the effect of PrP^C^-Apt DOA on the mitochondrial functions, proliferation, and apoptosis of CRC cells. Compared to free Dox, PrP^C^-Apt DOA significantly inhibited the mitochondrial function of CRCs. PrP^C^-Apt DOA also markedly inhibited the proliferation of CRCs and induced apoptosis. In developing cancer targeted DDSs, selecting an appropriate cancer target is important [31]. Until now, EGFR, HER2, folate receptor, and transferrin receptor have been mainly used as cancer targets [31]. In this study, we demonstrated that an effective cancer-targeted DDSs can be developed by targeting PrP^C^. 

## 2. Results

### 2.1. Characterization of PrP^C^-Apt DOA

**Figure 1:**
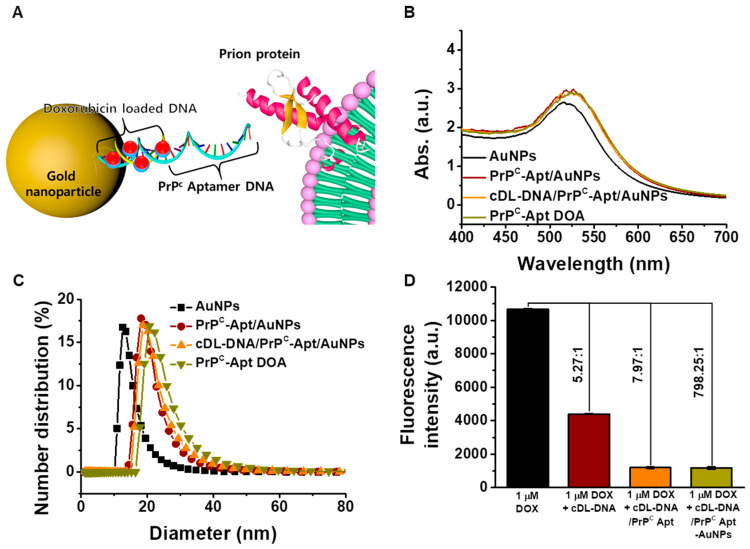
Synthesis and characterization of PrP^C^-Apt-functionalized doxorubicin-oligomer-AuNPs (PrP^C^-Apt DOA). (**A**) Schematic diagram of PrP^C^-Apt DOA. (**B**) UV-Vis spectra of AuNPs at each experimental step (*n* = 3). (**C**) dynamic light scattering (DLS) measurements for the hydrodynamic radius of PrP^C^-Apt DOA at each experimental step (*n* = 3). (**D**) Fluorescence measurement for the binding ratios of Dox to cDL-DNA, cDL-DNA/PrP^C^-Apt, and cDL-DNA/PrP^C^-Apt/AuNPs (*n* = 3).

The AuNPs were synthesized by the thermal reduction in AuCl_4_^−^ using citrate. To functionalize the AuNPs for PrP^C^ targeting, thiol-terminated PrP^C^-Apt was conjugated to the AuNPs. The complementary DNA was hybridized for Dox loading. Finally, Dox was loaded onto the AuNPs to generate PrP^C^-Apt DOA (Figure 1A). The size of AuNPs is one of the important parameters that affects drug loading, cellular uptake, and *in vivo* targeting [32,33]. Because AuNPs have intrinsic self-aggregation properties, they may gradually increase in size or become crystallized over time [34]. Therefore, it is important to characterize AuNPs during preparation. A transmission electron microscopy image of AuNPs revealed that the AuNPs have spherical shapes with 13 nm of diameter suggesting successful synthesis of AuNPs (Supplemental Figure S1). Ultraviolet-visible (UV-Vis) spectroscopy and dynamic light scattering (DLS) analyses were conducted to characterize the size of PrP^C^-Apt DOA in the present study. Figure 1B shows the absorption spectra of AuNPs at each preparation step of PrP^C^-Apt DOA. All absorption spectra of AuNPs showed a similar shape and λ_max_ of 520 nm, which indicated that the AuNPs had a diameter of 13 nm without aggregation, owing to electrostatic repulsive forces [35,36]. DLS analysis was conducted to measure the size distribution of PrP^C^-Apt DOA. As shown in Figure 1C, the average diameter of bare AuNPs was 13 nm and that of PrP^C^-Apt-conjugated AuNPs increased to 20 nm after the incubation of the bare AuNPs with the aptamers, suggesting successful immobilization of PrP^C^-Apt onto the AuNPs (Figure 1C). As Dox has an intrinsic fluorescence and quenching effect by photoelectron transfer (PET) from the π-electrons of intercalated DNA, the Dox loading amount of Apt-DOA was determined using fluorescence spectroscopy. The amount of Dox loaded onto complementary drug loading DNA (cDL-DNA), cDL-DNA/PrP^C^ Apt, and cDL-DNA/PrP^C^ Apt-AuNPs were determined to be 5.27, 7.97, and 798.25 molar equivalents (Dox/cDL-DNA, Dox/cDL-DNA/PrP^C^ Apt, Dox/cDL-DNA/PrP^C^ Apt-AuNPs), respectively (Figure 1D). Furthermore, we determined the fluorescence intensity of Dox which released from PrP^C^-Apt DOA in different conditions of pH and temperature. The initial-burst release was observed within 2 h in all the conditions, though the amount of Dox in pH 5.4 released was higher than pH 7.4. The fluorescence intensity of Dox showed negligible changes in different temperature condition of 25 °C and 36.5 °C (Supplemental Figure S2). These results indicate the possibility of a pH-triggered drug release of the PrP^C^-Apt DOA due to the acidic pH condition of the cancer microenvironment. Taken together, these results suggest that PrP^C^-Apt DOA are stable spherical nanoparticles, without aggregation or degradation. In addition, PrP^C^-Apt DOA serve as a promising DDS with a high drug-loading capacity, which is capable of loading a large amount of Dox.

### 2.2. PrP^C^-Apt DOA Show Active Targeting to PrP^C^-Positive CRC Cells

**Figure 2:**
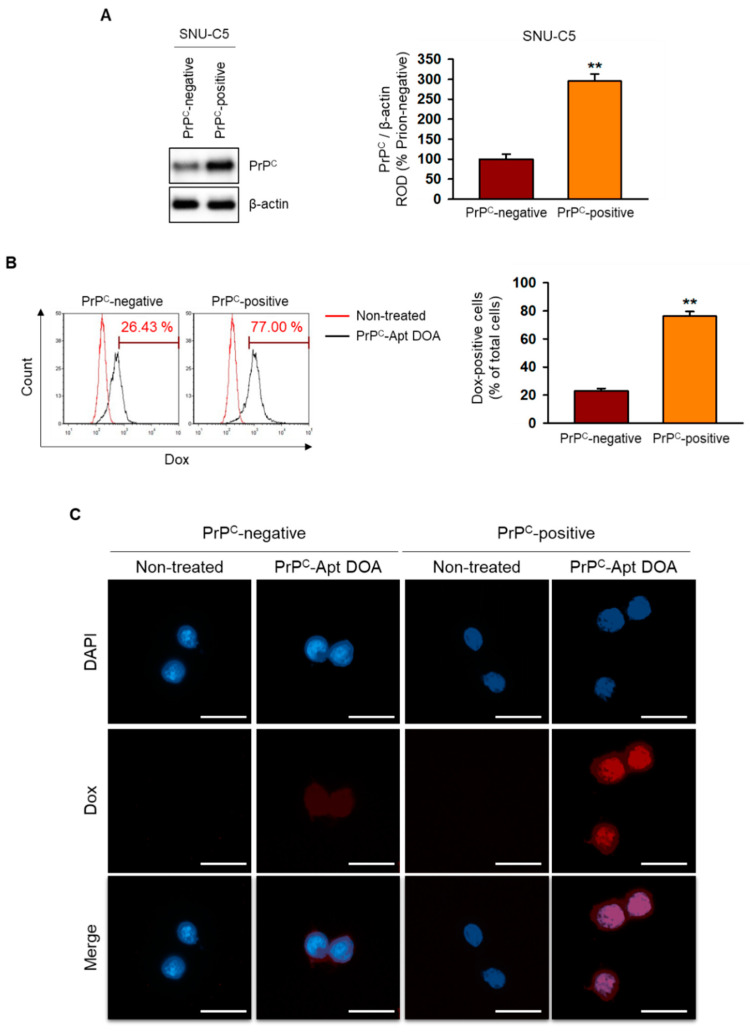
PrP^C^ targeting of PrP^C^-Apt DOA. (**A**) Expression levels of PrP^C^ were determined in PrP^C^-negative and PrP^C^-positive cells (*n* = 3). (**B**,**C**) Cellular uptake of PrP^C^-Apt DOA in PrP^C^-negative and PrP^C^-positive cells were determined by using flow cytometry (**B**) and fluorescence microscopy (**C**) (*n* = 3). Values represent mean ± SEM. ** *p* < 0.01 vs. PrP^C^-negative.

Next, we checked the active targeting of PrP^C^-Apt DOA to PrP^C^-positive CRC cells. First, we separated the PrP^C^-negative cells and PrP^C^-positive cells from SNU-C5 CRC cells using magnetic activated cell sorting. We determined the expression of PrP^C^ in PrP^C^-negative and PrP^C^-positive cells and confirmed that PrP^C^ expression is higher in the PrP^C^-positive cells (Figure 2A). Then, we evaluated PrP^C^ targeting of PrP^C^-Apt DOA after the treatment of PrP^C^-Apt DOA to PrP^C^-negative and PrP^C^-positive cells. The flow cytometry results revealed that the uptake of PrP^C^-Apt DOA was significantly increased in PrP^C^-positive cells compared to PrP^C^-negative cells, suggesting the interaction of PrP^C^ Apt and PrP^C^ facilitates uptake of PrP^C^-Apt DOA (Figure 2B). We further confirmed the PrP^C^ targeting of PrP^C^-Apt DOA using fluorescence microscopy. The results showed that the fluorescence intensity of Dox was greatly increased in PrP^C^-positive CRC cells. These results indicate that PrP^C^-Apt DOA can efficiently deliver Dox by targeting PrP^C^.

### 2.3. PrP^C^-Apt DOA Increase Reactive Oxygen Species (ROS) Formation by Decreasing Catalase and Superoxide Dismutase (SOD) Activities

**Figure 3:**
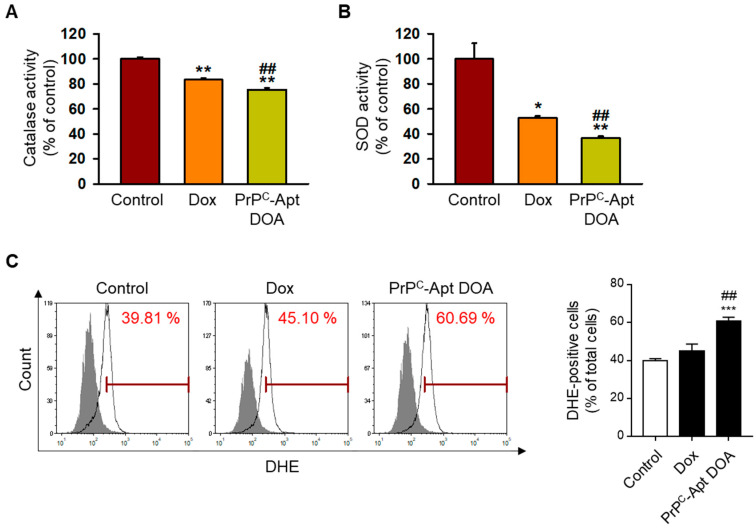
PrP^C^-Apt DOA induce more Reactive Oxygen Species (ROS) generation in colorectal cancer CRC cells than free Dox. (**A**) Catalase activity (*n* = 3) and (**B**) Superoxide Dismutase (SOD) activity (*n* = 3) in SNU-C5 cells post treatment with free Dox and PrP^C^-Apt DOA. (**C**) dihydroethidium (DHE) staining of SNU-C5 cells, as measured using flow cytometry (*n* = 3). The percentages of DHE-positive cells are shown in the graph. Values represent mean ± SEM (*n* = 3). * *p* < 0.05, ** *p* < 0.01, *** *p* < 0.001 vs. control; ## *p* < 0.05 vs. free Dox.

As Dox treatment is known to induce ROS formation, we first determined the effect of PrP^C^-Apt DOA on ROS formation. To determine whether PrP^C^-Apt DOA inhibit the catalase activity of CRC cells, SNU-C5 cells were treated with PrP^C^-Apt DOA, followed by measurement of the catalase activity. As shown in Figure 3A, treatment of SNU-C5 cells with PrP^C^-Apt DOA (2.5 nM of PrP^C^-Apt DOA containing 2 μM of Dox, 48 h) caused a greater decrease in the catalase activity, as compared to treatment with an equivalent concentration of free Dox (2 μM, 48 h). To further determine the effect of PrP^C^-Apt DOA on SOD activity, SNU-C5 cells were treated with PrP^C^-Apt DOA or free Dox, and the SOD activity was measured. As expected, treatment with PrP^C^-Apt DOA caused a greater decrease in the SOD activity of SNU-C5 cells, as compared to an equivalent concentration of free Dox (Figure 3B). Finally, we performed flow cytometry analysis of PrP^C^-Apt DOA or free Dox-treated SNU-C5 cells post dihydroethidium (DHE) staining. Since DHE is oxidized by ROS and become fluorescent, the ROS generation in live cells can be represented as total DHE fluorescence. DHE staining results showed that PrP^C^-Apt DOA significantly increased ROS formation in SNU-C5 cells (Figure 3C). These results indicate that PrP^C^-Apt DOA efficiently increased ROS formation in CRC cells by decreasing catalase and SOD activities.

### 2.4. PrP^C^-Apt DOA Efficiently Inhibit the Mitochondrial Function of CRC Cells

**Figure 4:**
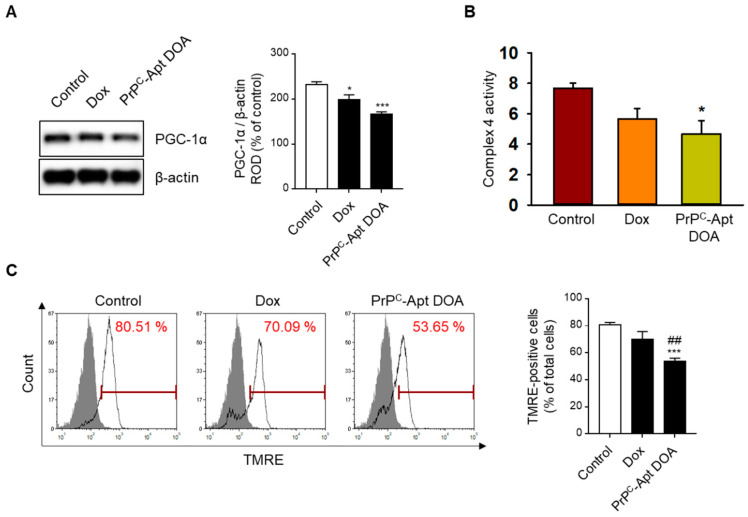
PrP^C^-Apt DOA reduce PGC-1α expression and mitochondrial membrane potential. (**A**) Expression levels of PGC-1α were determined post treatment of SNU-C5 cells with free Dox or PrP^C^-Apt DOA (*n* = 3). (**B**) Complex IV activity was analyzed post treatment of SNU-C5 cells with free Dox or PrP^C^-Apt DOA (n = 3). (**C**) The tetramethylrhodamine ethyl ester (TMRE)-positive population was quantified using flow cytometry analysis post treatment of SNU-C5 cells with free Dox or PrP^C^-Apt DOA (*n* = 3). Values represent mean ± SEM. * *p* < 0.05, ** *p* < 0.01, *** *p* < 0.001 vs. control; ## *p* < 0.01 vs. free Dox.

**Figure 5:**
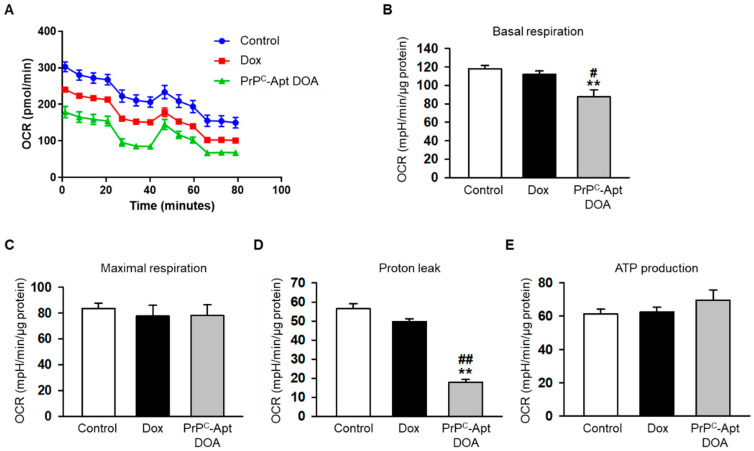
PrP^C^-Apt DOA inhibit mitochondrial function of CRC cells to a greater degree, as compared to free Dox. (**A**) Mitochondrial oxygen consumption rates (OCRs) of SNU-C5 cells treated with free Dox or PrP^C^-Apt DOA were measured over time (min). The cells were treated with oligomycin (1 μM, 20 min), FCCP (0.75 μM, 40 min), and antimycin A (1 μM, 60 min) to obtain bioenergetic parameters. (**B**) Basal respiration was measured by subtracting non-mitochondrial OCR (which is the remaining OCR post antimycin A treatment) from the initial OCR level. (**C**) Maximal respiration was measured upon addition of FCCP. (**D**) Proton leak was calculated by subtracting the OCR post antimycin A treatment from the OCR post oligomycin A treatment. (**E**) Adenosine triphosphate (ATP) production was determined by subtracting non-mitochondrial OCR from the OCR value of basal respiration. The histogram shows representative data from one replicate experiment (*n* = 5). The values represent mean ± SEM. ** *p* < 0.01 vs. control; # *p* < 0.05, ## *p* < 0.01 vs. free Dox.

To determine whether PrP^C^-Apt DOA affects the mitochondrial function of CRC cells, we performed western blot analysis of PrP^C^-Apt DOA-treated CRC cells for peroxisome proliferator-activated receptor gamma coactivator 1-alpha (PGC-1α) expression. PGC-1α is a transcriptional coactivator that regulates cellular energy metabolism. PGC-1α enhances mitochondrial function and promotes mitochondrial biogenesis [37]. Compared to treatment with free Dox, treatment with PrP^C^-Apt DOA further decreased the expression of PGC-1α in CRC cells (Figure 4A). Next, we determined the effect of PrP^C^-Apt DOA on the activity of mitochondrial complex IV and the mitochondrial membrane potential. As expected, treatment with PrP^C^-Apt DOA significantly decreased the complex IV activity and tetramethylrhodamine ethyl ester (TMRE)-positive population in CRC cells (Figure 4B,C). Next, we further confirmed the effect of PrP^C^-Apt DOA on mitochondrial function by measuring the oxygen consumption rate (OCR) of the CRC cells. Treatment with PrP^C^-Apt DOA significantly decreased basal respiration and proton leak in the mitochondria of CRC cells, with no effect on maximal respiration and ATP production (Figure 5A–E). These results suggest that PrP^C^-Apt DOA efficiently delivered Dox into the CRC cells, decreased PGC-1α expression, and inhibited the mitochondrial function of CRC cells.

### 2.5. PrP^C^-Apt DOA Efficiently Inhibit Proliferation of CRC Cells in Addition to Promoting Apoptosis

**Figure 6:**
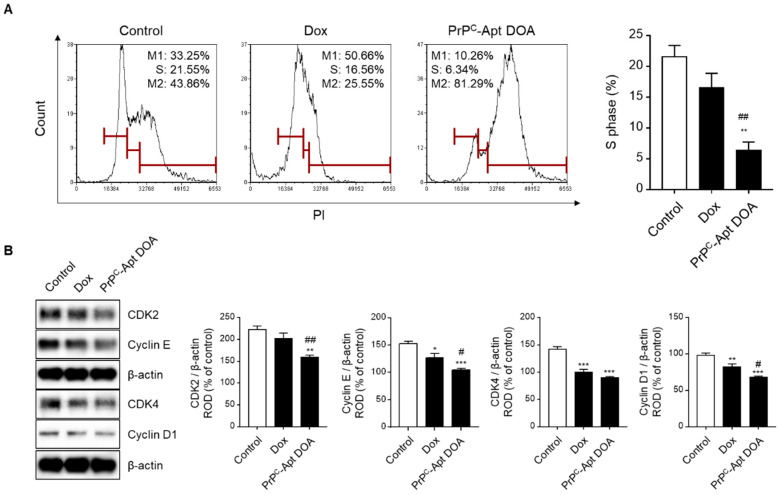
PrP^C^-Apt DOA efficiently inhibit the proliferation of CRC cells to a greater extent than free Dox. (**A**) Cell cycle analysis of SNU-C5 cells post treatment with free Dox and PrP^C^-Apt DOA (*n* = 3). (**B**) Western blot analysis of SNU-C5 cells for CDK2, cyclin E, CDK4, and cyclin D1 post treatment with free Dox and PrP^C^-Apt DOA (*n* = 3). Protein levels were determined using densitometry relative to β-actin. The values represent mean ± SEM. * *p* < 0.05, ** *p* < 0.01, *** *p* < 0.001 vs. control; # *p* < 0.05, ## *p* < 0.01 vs. free Dox.

**Figure 7:**
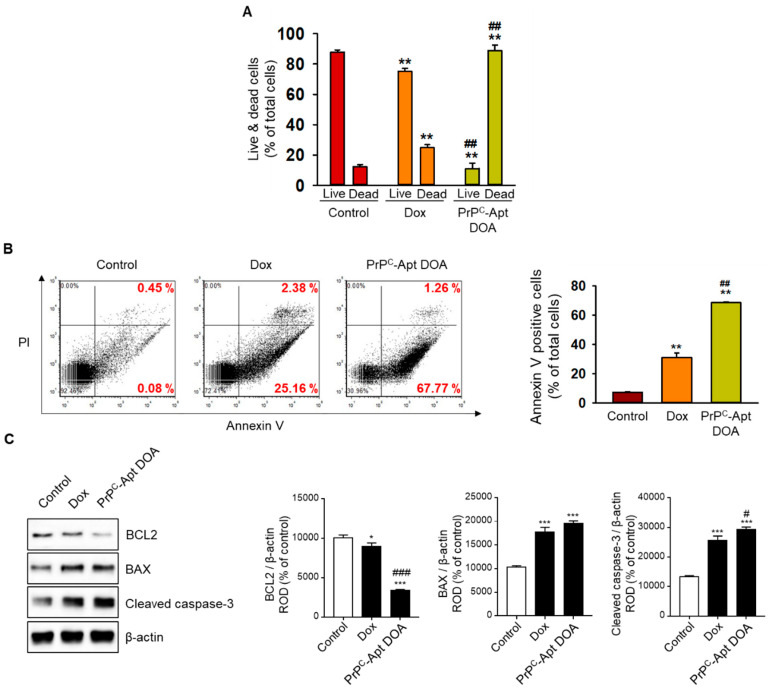
PrP^C^-Apt DOA efficiently promote the apoptosis of CRC cells. (**A**) Live and dead cells assay in SNU-C5 cells post treatment with free Dox and PrP^C^-Apt DOA. (**B**) Annexin V/PI staining of SNU-C5 cells post treatment with free Dox and PrP^C^-Apt DOA. (**C**) Western blot analysis of apoptosis-related proteins in SNU-C5 cells post treatment with free Dox and PrP^C^-Apt DOA. The values represent mean ± SEM. * *p* < 0.05, ** *p* < 0.01, *** *p* < 0.001 vs. control; # *p* < 0.05, ## *p* < 0.01, ### *p* < 0.001 vs. free Dox.

To determine the effect of PrP^C^-Apt DOA on the proliferation of CRC cells, we performed cell cycle analysis using flow cytometry. The results revealed that PrP^C^-Apt DOA significantly decreased the percentage of cells in S-phase from 21.6% to 6.3%, while free Dox caused only a slight decrease in the same (from 21.6% to 16.6%) (Figure 6A). To further determine whether PrP^C^-Apt DOA inhibit the proliferation of CRC cells, western blot analysis was performed for cell cycle-associated proteins, such as cyclin-dependent kinase (CDK) 2, cyclin E, CDK4, and cyclin D1. Treatment with PrP^C^-Apt DOA resulted in a greater decrease in the expression of cell cycle-related proteins, as compared to treatment with free Dox (Figure 6B). To determine whether treatment with PrP^C^-Apt DOA increased the number of dead cells in CRC cells, live and dead cells were quantified in free Dox- or PrP^C^-Apt DOA-treated SNU-C5 cells. As shown in Figure 7A, treatment with PrP^C^-Apt DOA significantly increased the percentage of dead cells, compared to treatment with free Dox (Figure 7A). Next, Annexin V/propidium iodide (PI) flow cytometry analysis was performed to investigate whether PrP^C^-Apt DOA increase apoptosis in CRC cells. Treatment with PrP^C^-Apt DOA further increased the percentage of early and late apoptosis to 67%, compared to that in the free Dox-treated group (27%) (Figure 7B). Consistent with these results, western blot analysis demonstrated that treatment with PrP^C^-Apt DOA further decreased the expression of B-cell lymphoma 2 (BCL2) and increased that of Bcl-2-associated X protein (BAX) and cleaved caspase-3, as compared to that with free Dox treatment (Figure 7C). Taken together, these results suggest that PrP^C^-Apt DOA effectively inhibited proliferation and increased apoptosis by efficiently delivering Dox to CRC cells by targeting PrP^C^.

### 2.6. PrP^C^-Apt DOA Efficiently Inhibit the Sphere Formation Capacity of CRC Cells

**Figure 8:**
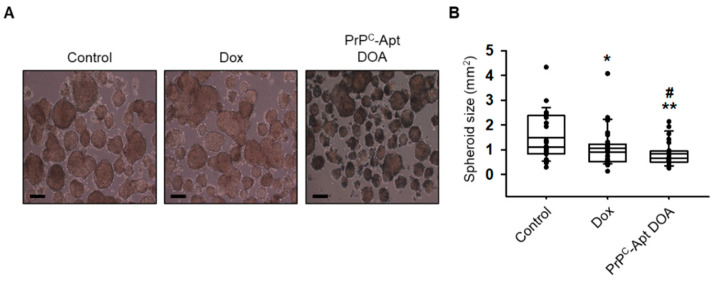
PrP^C^-Apt DOA efficiently inhibit the sphere formation capacity of CRC cells. (**A**) Sphere formation assay of SNU-C5 cells post treatment with free Dox or PrP^C^-Apt DOA (*n* = 3). Scale bar: 200 μm. (**B**) Diameters of the spheroids are shown in the graph. Dot symbols represent individual values. The values represent mean ± SEM. * *p* < 0.05, ** *p* < 0.01 vs. control; # *p* < 0.05 vs. free Dox.

The sphere formation assay is often used to assess the stem cell properties of cancer cells. We determined whether PrP^C^-Apt DOA inhibit the sphere formation capacity of CRC cells. SNU-C5 cells were cultured in ultra-low attachment six-well plates for 14 d in the presence of free Dox or PrP^C^-Apt DOA. As shown in Figure 8A, while free Dox also inhibited sphere formation of CRC cells, PrP^C^-Apt DOA efficiently inhibited sphere formation in SNU-C5 cells, as compared to free DOX. The diameters of the spheres were significantly reduced in PrP^C^-Apt-DOA-treated SNU-C5 cells (Figure 8B). These results suggest that PrP^C^-Apt DOA not only increased the apoptosis of CRC cells, but also inhibited the cancer stem cell properties of CRC cells.

## 3. Discussion

In this study, we developed PrP^C^-targeting AuNPs as an anti-cancer DDS. PrP^C^-Apt-conjugated AuNPs synthesized in this study were spherical nanoparticles with an average diameter of 20 nm. PrP^C^-Apt AuNPs displayed efficient loading of Dox and PrP^C^-targeted delivery of Dox to CRC cells. PrP^C^-Apt DOA efficiently inhibited the function of mitochondria, cell proliferation, and induced apoptosis of CRC cells, as compared to free Dox. In addition, PrP^C^-Apt DOA efficiently decreased the sphere formation capacity of CRC cells. These results suggest that active targeted drug delivery to CRC can be achieved by targeting PrP^C^. Thus, PrP^C^-Apt DOA may replace oxaliplatin and 5-FU as effective therapeutic agents that can be used in CRC treatment.

Compared to other nano-based DDSs, such as liposomes, iron oxide nanoparticles, and quantum dots, AuNPs have several advantages as DDSs; specifically, AuNPs are capable of being synthesized in different sizes and shapes, and allow for ease of surface modification. In addition, AuNPs can be loaded with various types of drugs, including chemical drugs [38,39,40], oligonucleotides [41], proteins [42], and vaccines [43]. Furthermore, DNA-conjugated AuNPs can be used as pH-dependent targeted DDSs, as the G and C bases are depurinated under acidic conditions, leading to the structural transformation of double-stranded DNA and Dox. The surface of AuNPs can be modified with various molecules, such as PEG [44], peptides [45], antibodies [46], or aptamers [47,48] to enhance their stability or to functionalize the NPs for targeted delivery systems. The prostate-specific membrane antigen (PSMA)-targeting aptamer, A10, was conjugated to the surface of AuNPs, and these A10-conjugated AuNPs display high sensitivity and specificity towards PSMA-expressing prostate cancer cells [49]. In addition, Ramos cell aptamer-conjugated AuNPs display specific recognition of tumor sites [50]. In this study, we conjugated PrP^C^ aptamers to AuNPs for Dox delivery to CRC. The AuNPs efficiently delivered Dox to PrP^C^-positive CRC cells and induced ROS generation, mitochondrial damage, and apoptosis.

We developed Dox DDSs using PrP^C^ aptamer-functionalized AuNPs. Many similar studies on the development of AuNP-based Dox DDSs have been reported [51]. Ramalingam et al. developed Dox DDSs by loading Dox into polyvinylpyrrolidone (PVP)-stabilized AuNPs [52]. The Dox-loaded PVP-AuNPs induced increased ROS generation and apoptosis in lung cancer compared to free Dox and PVP-AuNPs without Dox. Similarly, Du et al. also developed AuNP-based Dox DDSs by directly conjugating thoiolated doxorubicin analogs to PEG-modified AuNPs [39]. The doxorubicin analogs contain glutathione-reactive moiety, which facilitates Dox release under a reducing tumor environment. Although these Dox DDSs showed efficient Dox loading and anticancer efficacy, they lack cancer targeting ligands, which may reduce cancer-specific delivery. Many studies on active targeting of drug-loaded AuNPs using cancer targeting ligands such as antibodies, peptides, and small molecules also have been reported [53]. Zhang et al. developed 5-fluorodeoxyuridine (FUdR) and Dox co-delivery systems using HER2-specific affibody-functionalized AuNPs [54]. They conjugated FUdR containing DNA to HER2-specifc affibody and attached the affibody-DNA hybrid to AuNPs. However, displaying antibodies or small molecules on the surface of AuNPs requires additional synthetic reactions, which may complicate the development process and increase batch-to-batch variation and difficulty in mass production. 

Aptamers have several advantages as cancer targeting ligands since they are easy to synthesize, stable, non-immunogenic, and low-cost. A recent review found that EpCAM and Mucin-1 have been the most popular aptamer targets for CRC [55]. Mashreghi et al. developed CRC-targeted Dox DDSs using an SYL3C aptamer (which targets EpCAM)-functionalized liposomes [56]. Moosavian et al. utilized the 5TR1-aptamer, which targets Mucin1, for the development of PEGylated liposomal Dox DDSs [57]. However, the *K*_d_ value of SYL3C aptamer and 5TR1 aptamer are known as 38 nM [58] and 21 nM [59], respectively, which is higher than that of PrP^C^ aptamer of 16 nM [60]. Although PrP^C^ is overexpressed in CRC, and PrP^C^ aptamer is promising for CRC targeting, the development of CRC-targeted DDSs using PrP^C^ aptamer has been lacking. Du et al. developed the AuNPs-DNA (Dox) which is a Dox delivery system using PrP^C^ aptamer-functionalized AuNPs [61]. The AuNPs-DNA (Dox) looks similar to our PrP^C^-Apt DOA in that it is composed of AuNPs, PrP^C^ aptamer, and Dox. However, AuNPs-DNA (Dox) and PrP^C^-Apt DOA show differences in terms of DNA structure. In AuNPs-DNA (Dox), Dox is loaded at double-strand CG sequences in the stem region and the PrP^C^ aptamer is located at the loop region. Upon binding of PrP^C^ aptamer to PrP^C^, double-strand DNA structure is destroyed and Dox are released from DNA, which may reduce drug delivery efficiency. However, in PrP^C^-Apt DOA, even if the PrP^C^ aptamer binds to PrP^C^, it does not affect the structure of double-strand DNA and the Dox release. In addition, AuNPs-DNA (Dox) is used for human bone marrow neuroblastoma, whereas PrP^C^-Apt DOA is used for CRC cells.

Although PrP^C^-Apt DOA is an excellent DDS for CRC, it is necessary to improve PrP^C^-Apt DOA compared to other DDSs. PrP^C^-Apt DOA can be loaded with other drugs such as photosensitizers [62] and therapeutic nucleic acids [63] to develop co-delivery systems, which would show better therapeutic effects for Dox-resistant CRC. In addition, by changing the shapes, sizes and organizations of PrP^C^-Apt DOA, we may improve plasmonic photothermal properties and use them as phototheramal agents [64].

AuNPs are in the spotlight as promising DDSs since they are easy to synthesize, have excellent stability, and have the potentials to be used in various biomedical applications [65]. However, at the same time, the toxicity of AuNPs is being raised as an important issue [66]. Factors involved in the toxicity of AuNPs include size and shape, and surface chemistry of AuNPs [66,67]. In particular, it is known that the toxicity of AuNP can be significantly reduced through surface modification. In general, PEG is widely used to reduce the toxicity of AuNPs [22,68]. Patlolla et al. showed that the PEG coating of AuNPs reduced the ROS generation and hepatotoxicity compared to uncoated AuNPs [69]. In our previous study, DNA-conjugated AuNPs did not show toxicity in three human ovarian cancer cell lines, SK-OV-3, HEY A8, and A2780 [19]. However, further studies are needed to minimize the toxicity of AuNP by modifying the size, shape, and surface chemistry to promote applications of AuNPs in biomedicine.

In conclusion, we developed a PrP^C^-targeted Dox DDS. We demonstrated that PrP^C^-Apt DOA efficiently delivered Dox to CRC cells and induced ROS generation, mitochondrial damage, and apoptosis, as compared to free Dox. These results suggest that PrP^C^-APT DOA can serve as an effective therapeutic agent for CRC treatment. Future studies need to evaluate the in vivo safety, targeting, and anti-tumor effects of Prion Apt-DOA in CRC xenograft models, to assess the potential of this DDS.

## 4. Materials and Methods

### 4.1. Materials

Gold(III) chloride trihydrate, Dox hydrochloride, sodium citrate tribasic dihydrate, DHE, and phosphate-buffered saline (PBS) were purchased from Sigma-Aldrich. Thiol-terminated PrP^C^ aptamer DNA (5′-HS-C6-AAAAAAAAAA-TCG-TCG-TCG-TCG-TCG-TCG-TCG-CGGTGGGGCAATTTCTCCTACTGT-3′, underlined sequence is for the PrP^C^ aptamer) and cDL-DNA (5′-CGA-CGA-CGA-CGA-CGA-CGA-CGA-3′) were obtained from GenoTech (Daejeon, Republic of Korea).

### 4.2. Synthesis of PrP^C^-Apt DOA

PrP^C^-Apt DOA were synthesized according to a previously reported protocol [18]. AuNPs were synthesized in a single phase using a thermal reduction method with a trisodium citrate dihydrate reduction agent. First, 1 mL of 38.8 mM sodium citrate dihydrate solution was added rapidly to 10 mL of 1 mM HAuCl_4_ boiling solution. The reaction was stopped when the color of the solution changed from yellow to deep red. It is known that the color of the reaction mixture changes from yellow to deep red during the synthesis. Gold nanoparticles larger than 2 nm in diameter exhibit the characteristic surface plasmon band and such particles appear deep red [70]. The AuNP solution was then cooled to room temperature with stirring. To modify the AuNPs with PrP^C^-Apt, 100 μL of 10 μM PrP^C^-Apt was added to 1 mL of 10 nM AuNPs. The mixed solution was then incubated in a shaking incubator at 350 rpm and 25 °C for 16 h. Before further incubation, 100 μL of 1 M sodium chloride and 100 μL of 0.1 M phosphate buffer were added to the mixed solution. After incubation, the mixed solution was purified by centrifuging the PrP^C^-Apt and AuNPs mixed solution at 13,500 rpm for 20 min. To hybridize PrP^C^-Apt and cDL-DNA, 100 μL of 10 μM cDL-DNA was added to the PrP^C^-Apt/AuNPs solution at 350 rpm, 95 °C for 5 min. The mixed solution was then incubated in a shaking incubator at 350 rpm and 25 °C for 1 h. The PrP^C^-Apt/cDL-DNA/AuNPs solution was then purified by centrifuging twice at 13,500 rpm for 20 min. Finally, for loading of PrP^C^-Apt/AuNPs with Dox, 100 μL of 100 μM Dox was added to 1 mL of PrP^C^-Apt/AuNPs and incubated in a shaking incubator at 350 rpm, 25 °C for 1 h. The mixed solution was then purified by centrifuging twice at 13,500 rpm for 20 min.

### 4.3. Characterization of PrP^C^-Apt DOA

The particle size of AuNPs at each experimental step was confirmed using a S-3100 UV-Vis spectrophotometer (Scinco Co. Ltd., Seoul, Korea) and a Zetasizer Nano ZS-90 DLS instrument (Malvern Panalytical, UK). The binding ratios of Dox to cDL-DNA, cDL-DNA/PrP^C^-Apt, and cDL-DNA/PrP^C^-Apt/AuNPs were measured using fluorescence spectroscopy with an FS-2 instrument (Scinco Co. Ltd.), at excitation and emission wavelengths of 480 and 592 nm, respectively. The loading capacity was calculated by methods used in our previous study [18]. For the drug loading capacity of PrP^C^-Apt DOA, the fluorescence of Dox was measured by fluorescence spectroscopy. Firstly, Dox was added into the 0.1 μM of cDL-DNA, cDL-DNA/PrP^C^-Apt, and cDL-DNA/PrP^C^-Apt+AuNP with 1 h of binding time. Then, the samples were purified by centrifugation at 13,500 rpm for 20 min twice.

### 4.4. Culture of Human CRC Cell Line

The human CRC SNU-C5 cells (available from Korea Cell Line Bank, 0000C5) were obtained from the Chosun University Research Center for Resistant Cells (Gwangju, Republic of Korea). The cells were grown in RPMI 1640 medium (Thermo Fisher Scientific, Waltham, MA, USA) containing 10% fetal bovine serum, L-glutamine, and antibiotics (Thermo Fisher Scientific) at 37 °C in a 5% CO_2_-humidified incubator.

### 4.5. DHE Staining

DHE was used to measure superoxide anion levels in PrP^C^-Apt DOA-treated SNU-C5 cells. The PrP^C^-Apt DOA-treated SNU-C5 cells were treated with 10 mM DHE for 30 min at 37 °C. DHE-positive cells were detected using a CyFlow^®^ Cube 8 instrument (Partec, Munster, Germany), after washing with PBS. FSC Express 5 software (De Novo Software, Los Angeles, CA, USA) was used for data analysis.

### 4.6. Measurement of Mitochondrial Membrane Potential

To measure the mitochondrial membrane potential of PrP^C^-Apt DOA or free Dox-treated (2 μM, 48 h) SNU-C5 cells, TMRE (Abcam, Cambridge, UK) was used to stain the cells. The PrP^C^-Apt DOA-treated or free Dox-treated SNU-C5 cells were trypsinized and then centrifuged. After washing with PBS, the cells were stained with 200 nM TMRE solution in PBS at 37 °C for 15 min. TMRE-positive cells were detected using flow cytometry (Sysmex, Kobe, Japan). 

### 4.7. Cell Cycle Analysis

SNU-C5 cells treated with free Dox or PrP^C^-Apt DOA (2 μM, 48 h) were collected and fixed with 70% ethanol at −20 °C for 2 h. After washing with cold PBS, the cells were treated with FxCycle™ PI/RNase staining solution (Thermo Fisher Scientific) at 4 °C for 1 h. Cell cycle analysis was carried out using flow cytometry (Sysmex). Events were recorded for at least 10^4^ cells per sample. The sample data were analyzed using FCS Express 5 software (DeNovo Software).

### 4.8. Catalase Activity Assay

For the measurement of catalase activity, 40 μg of whole cell lysates were treated with 20 mM H_2_O_2_ in 0.1 M Tris-HCl for 30 min. The sample was further treated with 50 mM Amplex Red reagent (Thermo Fisher Scientific) and 0.2 U/mL horseradish peroxidase (HRP) and incubated for 30 min at 37 °C. The decomposition of the substrate was measured using a microplate reader (Varioskan™ LUX, Thermo Fisher Scientific) at the wavelength of 563 nm with measurement time of 100 ms at 20 °C.

### 4.9. SOD Activity Assay

SNU-C5 cells were collected and lysed with RIPA buffer (Thermo Fisher Scientific). Whole cell lysates (total 50 μg of protein) were treated with SOD, an enzyme that catalyzes potentially harmful ROS in cells, and the signal was immediately measured, every minute for 15 min, at the wavelength of 450 nm using a microplate reader (Varioskan™ LUX). The measurement time was set at 100 ms and the measurement temperature was 20 °C.

### 4.10. Western Blotting

Whole cell lysates of free Dox- or PrP^C^-Apt DOA-treated SNU-C5 cells were separated using 8–12% SDS-PAGE and then transferred to PVDF membranes. Membranes were blocked using 3% skimmed milk for 1 h, followed by overnight incubation at 4 °C with primary antibodies against PGC-1α, CDK2, cyclin E, CDK4, cyclin D1, BCL2, BAX, cleaved caspase-3, and β-actin. After washing with TBST (with 0.05% Tween-20), the membranes were incubated with goat anti-rabbit IgG or goat anti-mouse IgG conjugated to HRP (Santa Cruz Biotechnology, Santa Cruz, CA, USA). The bands were detected using enhanced chemiluminescence (Amersham Pharmacia Biotech, England, UK). Mouse anti-PGC-1α antibody (NBP1-04676) was obtained from Novus Biologicals (Littleton, CO, USA). The mouse anti-CDK2 (SC-6248), mouse anti-cyclin E (SC-377100), mouse anti-CDK4 (SC-56277), mouse anti-cyclin D1 (SC-20044), mouse anti-BCL2 (SC-7382), rabbit anti-Bax (SC-6236), rabbit anti-cleaved caspase-3 (SC-7148), and mouse anti-β-actin (SC-47778) antibodies were purchased from Santa Cruz Biotechnology.

### 4.11. Mitochondrial Complex IV Activity Assays

Mitochondrial fractions isolated from free Dox- or PrP^C^-Apt DOA-treated SNU-C5 cells were incubated in the assay medium for 3 min. Complex IV activity was analyzed by adding 75 mL of cytochrome c previously reduced with sodium borohydride and measuring the absorbance at the wavelength of 550 nm. The assay medium was composed of 0.8 μmol/L antimycin, 50 μmol/L decylubiquinone, 1 mmol/L potassium cyanide, 50 mmol/L potassium phosphate, and 250 mmol/L sucrose, pH 7.4.

### 4.12. OCR Measurement

OCR was measured in SNU-C5 CRC cells using the XF96 Extracellular Flux Analyzer (Seahorse Bioscience, MA, USA). Briefly, SNU-C5 CRC cells were added at a density of 5.0 × 10^4^ cells/well into XF96 cell culture multi-well plates and incubated overnight at 37 °C in a 5% CO_2_-humidified incubator. XF calibrant was added to the XF96 cartridges, following which the cartridges were incubated overnight at 37 °C in a non-CO_2_ incubator. The cell growth medium was changed to XF medium and the cells were further incubated at 37 °C in a non-CO_2_ incubator for 1 h. Next, inhibitors, including oligomycin, FCCP, and antimycin A were diluted to the appropriate concentrations in XF medium and added into the corresponding microwells of the XF96 cartridge plates. Following equilibration of the sensor cartridges, the XF96 cell culture multi-well plates were loaded into the XF96 Extracellular Flux Analyzer for measurement of the OCR. OCR were measured every 7 min (after mixing for 3 min, waiting for 2 min, and measurement for 2 min), 3 times after injection of the respective compounds (1.5 μM oligomycin, 1 μM FCCP, and 0.5 μM Rotenone + Antimycin A). Seahorse Wave Desktop Software (Agilent Technologies, Santa Clara, CA, USA) was used for the analysis. 

### 4.13. Spheroid Culture

SNU-C5 CRC cells treated with free Dox or PrP^C^-Apt DOA were cultured in ultra-low attachment six-well plates (Corning, Corning, NY, USA) for spheroid formation. The CRC cells were incubated in RPMI 1640 medium and grown at 37 °C in a 5% CO_2_-humidified atmosphere. Spheroids were grown for 14 d and observed using an optical inverted microscope (Olympus, Tokyo, Japan).

## Supplementary Materials

The following are available online at https://www.mdpi.com/1422-0067/22/4/1976/s1.
