# Supplementary material for: PrPC Aptamer Conjugated–Gold Nanoparticles for Targeted Delivery of Doxorubicin to Colorectal Cancer Cells
Source: Int J Mol Sci. 2021 Feb 17;22(4):1976. doi: 10.3390/ijms22041976 (PMC7922473; doi:10.3390/ijms22041976)
Supplement: Supplementary file 1 [file ijms-22-01976-s001.pdf]

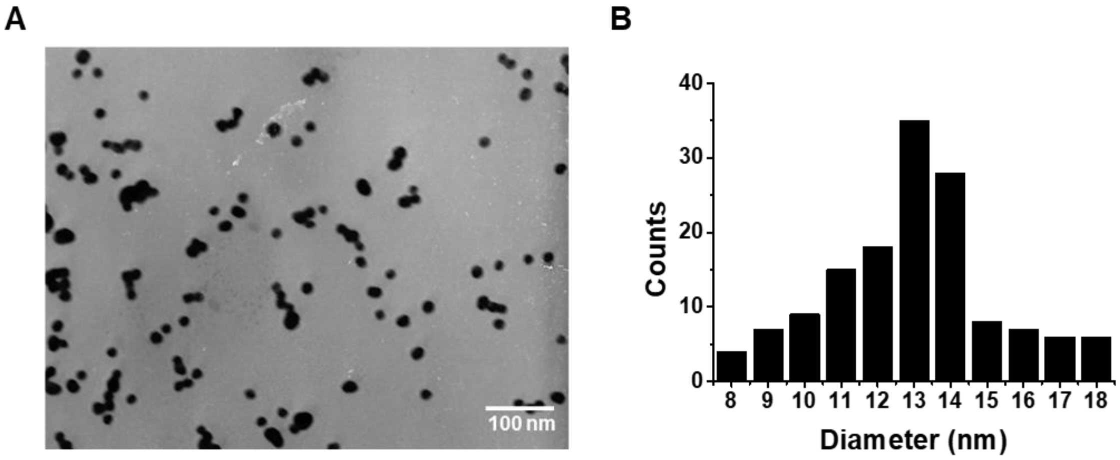

Supplemental Figure S1. TEM image and size distribution of the AuNPs. (A, B) The resulting TEM images (A) and size distribution (B) indicate the successfully synthesized AuNPs with spherical shapes and 13 nm of diameter.

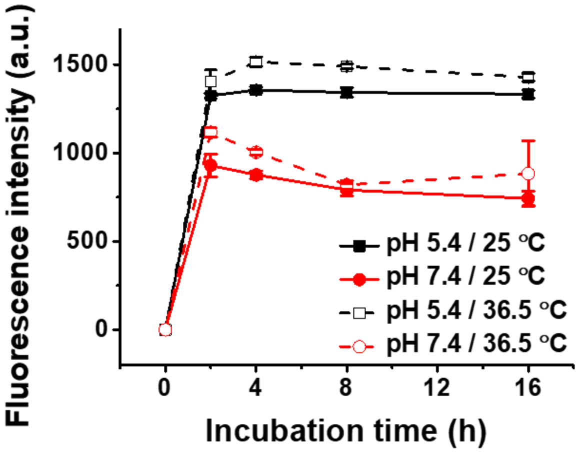

Supplemental Figure S2. Fluorescence intensity of Dox which released from PrP^{C}-Apt DOA in different conditions of pH and temperature. The initial-burst release was observed within 2 h in the all conditions, though the amount of Dox in pH 5.4 released higher than pH 7.4. The fluorescence intensity of Dox showed negligible changes in different temperature condition of 25 °C and 36.5 °C. These results indicate the possibility of pH-triggered drug release of the PrPc-Apt DOA due to the acidic pH condition of the cancer microenvironment.
